# Role of Double-Carbapenem Regimen in the Treatment of Infections due to Carbapenemase Producing Carbapenem-Resistant* Enterobacteriaceae*: A Single-Center, Observational Study

**DOI:** 10.1155/2018/2785696

**Published:** 2018-11-18

**Authors:** F. Cancelli, A. Oliva, M. De Angelis, M. T. Mascellino, C. M. Mastroianni, V. Vullo

**Affiliations:** Department of Public Health and Infectious Diseases, Sapienza University of Rome, Italy

## Abstract

**Purpose:**

(i) To compare infections caused by carbapenem-susceptible (CS) and carbapenemase producing carbapenem-resistant* Enterobacteriaceae* (CP-CRE); (ii) to evaluate the clinical effectiveness of the double-carbapenem (DC) regimen in comparison with the best available treatment (BAT) in infections caused by CP-CRE; and (iii) to determine the exact minimal inhibitory concentrations (MICs) of meropenem/ertapenem (MEM/ETP) and the degree of* in vitro* ETP+MEM synergism in subjects receiving the DC.

**Methodology:**

Over a 3-year period (2014-2017), patients with infections due to* Enterobacteriaceae* were included in a single-center, retrospective, observational study. According to the susceptibility to carbapenems, subjects were divided into CSE and CP-CRE groups. CP-CRE group was further divided into subjects receiving the DC regimen and those treated with other regimens (BAT group). Clinical characteristics and the presence of 5^th^-day response and 60-day outcome were evaluated for DC and BAT groups. The determination of MEM and ETP actual MICs and the MEM+ETP synergistic activity were performed on strains obtained from subjects receiving the DC regimen.

**Results:**

A total of 128 patients were included in the study: 55/128 (43%) with infections due to CP-CRE and 73/128 (57%) with infections due to CSE. Among CP-CRE (n=55), 21 subjects (39%) were treated with the DC regimen whereas 34 (61%) received BAT. No differences in terms of severity of infection, presence/absence of concomitant bacteremia, type of infection, and resolution of infection were found; in contrast, DC group tended to have a higher rate of sepsis or septic shock at the onset of infection and a higher rate of 5^th^-day response. MICs 50/90 were 256/512 and 256/256 *μ*g/mL for MEM and ETP, respectively. Overall, complete* in vitro* synergism was found in 6/20 strains (30%).

**Conclusion:**

The DC regimen is a valid and effective therapeutic option in patients with infections due to KPC producing CRE, including those with bacteremic infection and more severe clinical conditions. The clinical effectiveness is maintained even in the presence of extremely high MEM MICs.

## 1. Background

The rapid spread of multidrug-resistant bacteria has become a public health concern, especially in some countries where the spread of carbapenem-resistant microorganisms is endemic [1]. In particular, infections caused by CP-CRE are associated with a high treatment failure and consequent high mortality, given the limited therapeutic options and the lack of worldwide availability of new drugs such as ceftazidime/avibactam [2].

Risk factors for CP-CRE infections have been widely investigated and serve as possible drivers of prompting an appropriate antimicrobial therapy, aiming at improving the infection cure and reducing mortality [3].

Although the combination therapy is preferred over monotherapy, the optimal management of CP-CRE systemic infections remains a real challenge, which seems even more complicated given the emergence of resistance to ceftazidime/avibactam [4] and the rising diffusion of strains harbouring enzymes other than carbapenemases [1].

Recently, several efforts have been made with the aim of finding the most appropriate antimicrobial regimen according to the susceptibility profile of the microorganisms and the severity of infection [5, 6]. In this setting, the double-carbapenem regimen retains a place in therapy in patients with high risk of mortality, pan-drug resistant organisms, and lack of therapeutic options [5].

Based on these premises, aims of the study were (i) to compare infections caused by CSE with those caused by CP-CRE, (ii) to evaluate the clinical effectiveness of the DC regimen in comparison with BAT in infections caused by CP-CRE, and (iii) to determine the exact MICs of MEM/ETP and the degree of* in vitro* ETP+MEM synergism in subjects receiving the DC.

## 2. Materials and Methods

### 2.1. Study Population

This was a single-center, retrospective, observational study including patients hospitalized over a 3-year period (2014-2017), at the Department of Public Health and Infectious Diseases (Sapienza University, Rome) with infections due to* Enterobacteriaceae*.

Carbapenem susceptibility was determined using VITEK-2 system and interpreted in accordance with EUCAST breakpoint [6] whereas CP-CRE were defined following CDC case definition [7].

**Figure 1:**
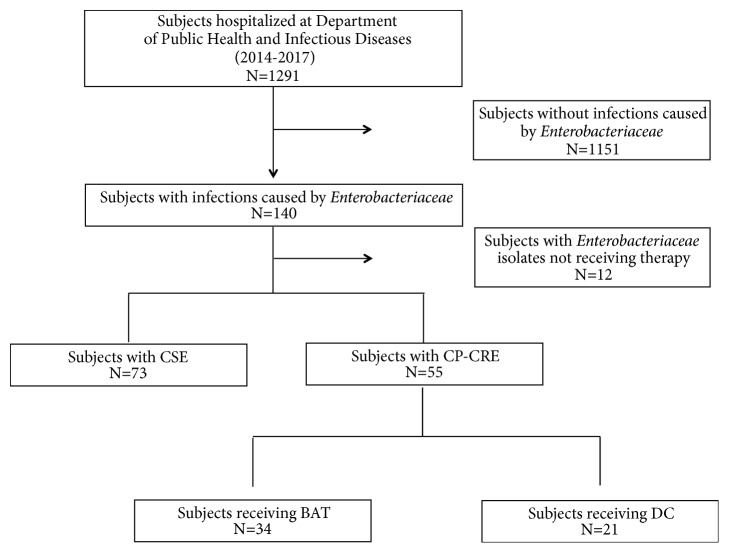
Flow chart of the study. CSE: carbapenem-susceptible* Enterobacteriaceae*; CP-CRE: carbapenemase producing carbapenem-resistant* Enterobacteriaceae*; DC: double-carbapenem; BAT: best available treatment.

Accordingly, subjects were divided into 2 groups: CP-CRE and CSE. CP-CRE group was further divided into subjects receiving the DC regimen and those treated with other regimens, defined as BAT group (Figure 1).

The DC consisted of ertapenem (1 g/day) followed by high doses of meropenem (6 g/day) or modified according to creatinine clearance. BAT was defined as the definitive therapy chosen by the Infectious Diseases specialists according to susceptibility profile of the microorganisms and the clinical conditions of the patients.

Demographic, clinical, and laboratoristic parameters were collected for each subject. Inclusion criteria were age >18 and patients with infections due to* Enterobacteriaceae* receiving antimicrobial therapy. Isolates collected from all sites of infection were also included. However, in case of multiple cultures from the same patient, only the first isolate causing infection was considered in the study. Exclusion criteria were age <18 and pregnancy.

Given the unconventionality of the treatment, all study participants receiving the DC regimen gave informed written consent. The study was approved by the local Ethics Committee.

### 2.2. Definitions

The clinical presentation of infection (sepsis, sepsis shock) was defined in accordance with the international guidelines [8].

The clinical and/or microbiological response at day 5 was defined as resolution of signs and symptoms of infections (i.e., defervescence, improvement of clinical conditions and imaging upon antimicrobial treatment) and/or negativity of cultures performed after 5 days of antimicrobial treatment, respectively, and expressed as a nominal variable. In addition, time to clinical response was defined as time (days) to resolution of fever and improvement in clinical or radiological status, expressed as a continuous variable. As for outcomes, clinical cure was defined as survival at 60 days, resolution of signs and symptoms of infection, and absence of recurrence at 60 days following the onset of infection [9]. Infection relapse was defined as recrudescence of infection after an initial response [10].

### 2.3. Microbiological Studies

The antimicrobial susceptibility pattern of* Enterobacteriaceae *was obtained through the VITEK-2 system (bioMerieux, Marcy l'Etoile, France).

Strains obtained from subjects receiving the DC regimen underwent additional microbiological analyses, including the phenotypic determination of carbapenemases [11]. The determination of meropenem and ertapenem actual MICs was obtained by the macrobroth dilution method [12] whereas the synergistic activity of meropenem plus ertapenem was performed by the checkerboard method and the fractional inhibitory concentration index (FICI) calculation. Briefly, a 96-well microtitre plate containing antibiotic combinations at different concentrations and a final inoculum of ~5 x 10^5^ CFU/ml of CP-CRE was incubated at 37°C for 24h under static conditions in Mueller Hinton Broth. The FICI of each combination was defined as follows: ∑FIC: FICA + FICB= MICA+B/MICA alone + MICB+A/MICB alone. A FICI ≤0.5 indicated synergism [13]. Experiments were performed in triplicate and the results were averaged.

### 2.4. Statistical Analysis

Results were expressed as mean ± standard deviation (SD) or median (range) and as percentages for continuous and categorical variables, respectively. Categorical variables (such as clinical and/or microbiological response at day 5) were compared by using the X^2^ or Fisher's exact tests, as appropriate, whereas continuous data (such as time to clinical response) were analyzed with Student's t-test and the nonparametric Mann–Whitney test. Statistical analyses were performed using STATA 9 software (STATA Corp. LP, College Station, Texas, USA) and GraphPad Prism version 7 for Windows (Graphpad Software MacKiev), as appropriate. All statistic tests were 2-tailed and a* p value* <0.05 was considered statistically significant.

## 3. Results

### 3.1. Carbapenemase Producing Carbapenem-Resistant vs Carbapenem-Susceptible Infections

A total of 128 patients were included in the study: 55/128 (43%) with infections due to CP-CRE and 73/128 (57%) with infections due to CSE (Table 1). Although not statistically significant, length of hospitalization before the onset of infection was higher in CP-CRE than in CSE (median 29.5 vs 17 days, p=0.13).

Clinical presentation was more severe (sepsis and/or septic shock) in CP-CRE than in CSE [10/55 (18.1%) vs 1/73 (1.4%) for sepsis, p=0.0009, 1/55 (1.8%) vs 0/73 (0%) for septic shock, p=0.42, respectively].

Although the time for obtaining clinical response did not differ between the 2 groups (median 4.5 vs 4 days, p=0.16), patients in CP-CRE group tended to have a lower rate of 5^th^-day response than subjects in CSE group. Compared with CSE, the overall length of hospitalization and mortality were higher in CP-CRE [median 31 vs 16 days, p<0.0001 and 6/55, 10.9% vs 2/74, 2.7%, p=0.07, respectively], with a global lower rate of infection cure at 60-day follow-up (43/55, 78.2% vs 67/73, 91.8%, p=0.03).

With regard to bacterial species, all the CP-CRE were* K. pneumoniae* whereas among CSE 58 (79.5%) were* E. coli *and 15 (20.5%)* K. pneumoniae*.

### 3.2. Carbapenemase Producing Carbapenem-Resistant Infections: DC Regimen vs BAT

Among CP-CRE (n=55), 21 subjects (39%) were treated with the DC regimen, with 3 subjects having received colistin and/or aminoglycosides prior to switch to the DC for 2, 2, and 3 days, respectively. The remaining 34 (61%) received other regimens [colistin-based combinations: 14 (colistin plus carbapenems±a third* in vitro* active drug: 9; colistin plus tigecycline±a third* in vitro* active drug: 2; colistin monotherapy: 1; colistin plus gentamicin: 1; colistin plus rifampin: 1) and other colistin-free regimens: 20 (aminoglycosides monotherapy: 13; high doses of carbapenems plus aminoglycosides: 6; high doses of meropenem plus fluoroquinolones: 1)].

Patients treated with DC tended to have a more severe clinical presentation (sepsis and/or septic shock) [6/21 (28.6%) vs 4/34 (11.7%) for sepsis, p=0.16, 1/21 (4.8%) vs 0/21 (0%) for septic shock, p=0.38, respectively]. Bacteremic infections were 7/21 (33.3%) and 7/34 (20.5%, p=0.34) for DC and BAT groups, respectively. As expected, in the DC group, colistin and aminoglycosides resistance rates were higher than those found in BAT group [10/21 (47.6%) vs 6/34 (17.6%) and 8/21 (38.1%) vs 4/34 (11.7%), p=0.04, 0.01, respectively] (Table 2).

Although DC patients tended to have a higher 5^th^-day response rate [13/21 (61.9%) vs 14/34 (41.1%), p=0.13], with a shorter time to clinical response (median 3 vs 6 days, p=0.25), the infection cure at 60-days did not differ between the two groups [16/21 (76.1%) vs 27/34 (79.4%), p=0.73]. In particular, mortality was 2/21 (9.5%) vs 4/34 (11.7%, p=0.99). A total of 6 patients had a recurrence of infection, equally distributed between DC and BAT groups [3/21 (14.2%) vs 3/34 (8.8%), p=0.66] (Table 2).

### 3.3. Microbiological Analyses

Microbiological analyses were performed on strains collected from subjects receiving DC regimen (n=20; 1 strain was not available) and are represented in Table 3.

All the isolated CP-CRE harboured KPC enzymes, which is in accordance with the local epidemiology [14].

MICs 50/90 were 256/512 and 256/256 *μ*g/mL for meropenem and ertapenem, respectively. Overall, complete* in vitro* synergism (expressed as FICI ≤0.5) was found in 6/20 strains (30%).

Among subjects with meropenem MIC was ≤128 *μ*g/mL (n=7), which has been found as the best* in vitro* MIC value for predicting the highest activity of the DC [15] the clinical outcome at 60 days was cure or relapse in the totality of cases (5 cure, 2 relapse) whereas in patients with meropenem MIC >128 *μ*g/mL (n=13) death occurred in 2 cases and cure in 11 (Table 4).

## 4. Discussion

Infections caused by CP-CRE are characterized by a higher morbidity and mortality than those caused by carbapenem-sensitive strains [2]. Given the worldwide spread of CP-CRE and the growing emergence of resistance to antimicrobials such as colistin and aminoglycosides, which have been used as last resort drugs, there is a growing literature investigating the best therapeutical regimen according to prognostic scores and/or antimicrobial susceptibility pattern of the microorganisms [16].

Furthermore, new agents with activity against CP-CRE show preferential activity against certain type of carbapenemases [17] and unfortunately their availability is still restricted to some countries, with obvious therapeutic limitations. The recent use of drugs such as ceftazidime/avibactam led to the consideration that it might be considered as a valid option in the setting of CRE infection [17]; however, its use might be undermined by the emergence of resistance, especially in strains harbouring KPC-3 enzymes and even during treatment [18].

In these challenging scenarios, the double-carbapenem regimen has been proposed as a possible therapeutic option in selected cases [14, 19–21]. While there have been positive clinical outcomes studies with double-carbapenem use and while* in vitro* studies have demonstrated bactericidal activity with the combination, the exact mechanism of action is not fully understood [10, 22–26].

In the present study, all consecutive patients with infections caused by* Enterobacteriaceae* hospitalized at the Department of Public Health and Infectious Diseases (Sapienza University, Rome) over a 3-year period were included. Apart from the observed differences between CP-CRE and CSE infections, which confirmed the widely reported data in the literature regarding epidemiology (with* K. pneumoniae* being the most frequent CR-CPE), a more severe clinical presentation and a lower rate of infection cure in CP-CRE, we were able to analyze a consistent number of patients treated with the DC regimen in comparison with the BAT group. As a matter of fact, no differences in terms of severity of infection, presence/absence of concomitant bacteremia, type of infection, and resolution of infection were found; in contrast, subjects treated with the DC tended to have a higher rate of sepsis/septic shock at the onset of infection and a higher rate of 5^th^-day response. Taken together, these findings confirm that the DC regimen represents a valid therapeutic option when no other alternatives are possible, with a global high clinical cure, similar to that observed with the BAT. However, it should be pointed out that performing the source control (i.e., catheter/stent removal, abscesses drainage) whenever possible as part of infection treatment might have contributed to the overall observed high clinical cure. Of note, the presence of bacteremic infections in one-third of subjects receiving the DC regimen strengthens the clinical effectiveness of this therapeutic option, which seems to retain its efficacy even in the presence of high bacterial inoculum, typically characteristic of bloodstream infections.

All the CP-CRE strains were* K. pneumoniae*: since KPC represents the most widely spread carbapenemase in our country, the results on the efficacy of DC might be translated even against KPC producing* Enterobacteriaceae* other than* K. pneumoniae* (i.e.,* E. coli*) [23].

The results of the present study are in line with some recent investigations evaluating the clinical role of the DC regimen when no other options are available or after failure of first-line regimens [22] or in critically ill patients [27]. In the first study, the authors found a high clinical and microbiological success in a cohort of patients with complicated urinary tract infections (with or without secondary bacteremia), bloodstream infections, pneumonia, and external ventricular drainage infection [22] whereas in the second case-control study conducted in two Italian Intensive Care Units subjects receiving DC regimen presented with more severe clinical condition and had an improved 28-day mortality compared with those treated with standard regimen including colistin, tigecycline, or gentamicin [27].

Moreover, the efficacy of the DC regimen has been demonstrated in immunocompromised patients, including kidney transplanted patients [23, 28] and a patient after allogenic hematopoietic stem cell transplantation [29].

One of the strengths of the present research is represented by the additional microbiological studies performed on the strains collected from patients receiving the DC regimen. In fact, automated systems such as VITEK-2, by indicating high carbapenems MIC as >16 *μ*g/mL, are unable to determine the precise MIC of carbapenems and there are growing evidences supporting the concept that knowing the real MIC of meropenem might influence the therapeutic choice and the effectiveness of carbapenem-based combination [15, 30]. In particular, the DC appeared to be more effective* in vitro* if the meropenem MIC is ≤128 *μ*g/mL [15]. In the present study, the MICs 50/90 for meropenem and ertapenem were extremely high, with only 7/21 (33.3%) strains with meropenem MIC ≤128 *μ*g/mL; nevertheless, the overall clinical cure was as high as for strains exhibiting higher MICs. Interestingly, these results are similar to those found in a previous study [22] where the actual meropenem MIC, which was performed in 20/27 strains, was >256 *μ*g/mL in 5/20 (20%) strains, in the absence of clinical failure. Thus, the exact role of carbapenem MIC in predicting the DC clinical success should be better understood and deserves further investigations, together with the interaction with the patients' immune system.

## 5. Conclusions

In conclusion, we demonstrated that the DC regimen is a valid and effective therapeutic option in patients with infections due to KPC producing CRE, including those with bacteremic infection and more severe clinical conditions. The clinical effectiveness is maintained even in the presence of extremely high meropenem MIC.

## Figures and Tables

**Table 1:** General characteristics of study population, according to the carbapenem susceptibility of *Enterobacteriaceae. *CP*-*CRE: carbapenemase producing carbapenem-resistant* Enterobacteriaceae; *CSE: carbapenem-susceptible* Enterobacteriaceae*;COPD: chronic obstructive pulmonary disease; HA: hospital-acquired; CA: community-acquired. °: Only subjects with active urinary tract infection requiring antimicrobial therapy were included; §: defined as resolution of signs and symptoms of infections (i.e., defervescence, improvement of clinical conditions and imaging upon antimicrobial treatment) and/or negativity of cultures performed after 5 days of antimicrobial treatment, respectively. *∗∗*: sepsis and septic shock were defined according to international guidelines [8].

	CP-CRE	CSE	*p-value*
	(n=55)	(n=73)	
***General characteristics***			
Age (years), mean (± SD)	61.15 (± 15.4)	64.7 (± 19.5)	0.2595
M:F, n	36:19	48:25	1
Charlson comorbidity index, mean (±SD)	5.24 (± 2.97)	5.82 (± 3.5)	0.3223

***Comorbidity, n (%)***			
Cancer	20 (36.4)	22 (30.1)	0.5687
Chronic Kidney disease	10 (18.2)	14 (19.2)	1.0000
Diabetes mellitus	12 (21.8)	13 (17.8)	0.6543
Heart failure	24 (43.6)	24 (32.9)	0.2689
Liver disease	5 (9.1)	8 (11)	0,7769
COPD	5 (9.1)	10 (13.7)	0.4143

***Modality acquisition of infection, n***			
HA:CA	50:5	38:35	**< 0.0001**

***Risk factors***			
Hospitalization day before infection, mean (± SD), median	36.12 (± 27.6), 29.5	23.46 (±19.5), 17	0.1376
Hospitalization in the last year, n (%)	51 (92.7)	46 (63)	**0.0001**
Urinary catheter, n (%)	34 (61.8)	21 (28.7)	**0.0003**
Central venous catheter, n (%)	26 (47.3)	9 (12.3)	**< 0.0001**
Tracheostomy, n (%)	10 (18.2)	0 (0)	**0.0001**

***Previous antibiotic therapy (90 days), n (%)***			
Cephalosporins	8 (14.5)	7 (9.6)	0.4171
Penicillin	16 (29.1)	13 (17.8)	0.1418
Carbapenems	19 (34.6)	4 (5.5)	**0.0002**
Fluoroquinolones	18 (32.7)	15 (20.5)	0.2311
Colistin	8 (14.5)	2 (2.3)	**0.0190**

***Clinical presentation, n (%)***			
Sepsis^*∗∗*^	10 (18.1)	1 (1.4)	**0.0009**
Septic shock^*∗∗*^	1 (1.8)	0	0.4297

***Site of infection***			
Lung	12 (21.8)	8 (11)	**0.0005**
Urinary tract°	37 (62.3)	52 (71.2)	0.6995
Soft tissue	12 (21.8)	11 (15.1)	0.3588
Bacteremic infection	14 (25.5)	12 (16.4)	0.2680
Primary bacteremia	4 (7.3)	7 (9.6)	0.7566

***Type of Enterobacteriaceae, n (%)***			**< 0.0001**
*Escherichia coli*	0 (0)	58 (79.5)	
*Klebsiella pneumoniae*	55 (100)	15 (20.5)	

***Antibiotic resistance profile, n (%)***			
Carbapenem	55 (100)	0 (0)	**< 0.0001**
Fluoroquinolones	53 (96.4)	47 (64.4)	**< 0.0001**
Aminoglycosides	12 (21.8)	24 (32.9)	0.2333
Colistin	15 (27.3)	1 (1.4)	**< 0.0001**
Tigecycline	30 (54.5)	4 (5.4)	**< 0.0001**

***Therapy***			
Time to clinical response, days, mean (± SD), median	6.6 (± 4.65), 4.5	5.2 ± 4.04, 4	0.1674
5th day response^§^, n (%)	28 (50.9)	47 (64.4)	0.1487

***Length of hospitalization, mean (± SD)***	39.2 ± 29.5	20.4 ± 14.1	**< 0.0001**
***median***	31	16	

***Outcome***, n (%):			
Clinical cure	43 (78.2)	67 (91.8)	**0.0393**
Infection relapse	6 (10.9)	4 (5.4)	0.4297
Death	6 (10.9)	2 (2.7)	0.0739

**Table 2:** Comparison between subjects treated with the double-carbapenem regimen (DC) and those treated with the best available treatment (BAT). *∗*: two infections were present in some patients. °: only subjects with active urinary tract infections requiring antimicrobial therapy were included; §: defined as resolution of signs and symptoms of infections (i.e., defervescence, improvement of clinical conditions and imaging upon antimicrobial treatment) and/or negativity of cultures performed after 5 days of antimicrobial treatment, respectively. *∗∗*: sepsis and septic shock were defined according to international guidelines [8].

	**Group DC**	**Group BAT**	***p* value**
	**(n= 21)**	**(n= 34)**	
***Demographic characteristics***			
Age (years), mean (± SD)	62.28 (± 12.1)	61.18 (± 17)	0.7971
M:F	14:7	21:13	1
Charlson comorbidity index, mean (±SD)	5.14 (± 2.76)	5.39 (± 3.78)	0.7940

***Clinical presentation, n (%)***			
Sepsis^*∗∗*^	6 (28.6)	4 (11.7)	0.1619
Septic shock^*∗∗*^	1 (4.8)	0 (0)	0.3889

***Site of infection, n (%)***			
Lung	4 (19)	8 (23.5)	0.7466
Urinary tract°	11 (52.4)	26 (76.4)	0.0702
Soft tissue	7 (33.3)	5 (14.7)	0.1795
Bacteremic infection	7 (33.3)	7 (20.5)	0.3453

***Antibiotic resistance, n (%)***			
Fluoroquinolones	21 (100)	31 (91.1)	0.5157
Aminoglycosides	8 (38.1)	4 (11.7)	**0.0424**
Colistin	10 (47.6)	6 (17.6)	**0.0136**
Tigecycline	11 (52.4)	18 (52.9)	1.0000

***Therapy:***			
Time to clinical response, days, mean (± SD), median	5.5 (± 4.22), 3	7.3 (± 4.87), 6	0.2570
5th day response^§^, n (%)	13 (61.9)	14 (41.1)	0.1351

***Outcome, n*** (%):			
Clinical cure	16 (76.2)	27 (79.4)	0.7329
Infection relapse	3 (14.2)	3 (8.8)	0.6660
Death	2 (9.5)	4 (11.7)	0.9980

**Table 3:** Microbiological studies on strains isolated from patients treated with the double-carbapenem regimen and correlation with clinical outcome. MEM: meropenem; ETP: ertapenem. °: complete synergy was defined as FICI ≤ 0.5, indifference as FICI > 0.5–4.0, and antagonism as FICI > 4.0 [13]. *∗*: one strain was not available for additional microbiological studies. NA: not applicable.

***Pt***	***MIC MEM, VITEK-2*** **(** ***μ*** ***g/mL)***	***MIC ETP, VITEK-2*** **(** ***μ*** ***g/mL)***	***Actual MIC MEM (*** ***μ*** ***g/mL)***	***Actual MIC ETP (*** ***μ*** ***g/mL)***	***Synergism MEM+ETP***°	***Outcome***
1	>16	>16	256	256	complete	died

2	>16	>16	512	128	indifference	cured

3	>16	>16	512	256	complete	cured

4	>16	>16	512	256	indifference	cured

5	>16	>16	128	256	indifference	relapsed

6	>16	>16	128	256	indifference	cured

7	>16	>16	128	256	indifference	cured

8	>16	>16	256	256	complete	cured

9	>16	>16	32	64	indifference	cured

10	>16	>16	128	128	indifference	cured

11^*∗*^	>16	>16	NA	NA	NA	relapsed

12	>16	>16	256	128	indifference	cured

13	>16	>16	256	128	indifference	cured

14	>16	>16	256	256	indifference	cured

15	>16	>16	128	128	indifference	cured

16	>16	>16	256	256	indifference	died

17	>16	>16	512	512	indifference	cured

18	>16	>16	512	512	complete	cured

19	>16	>16	256	128	complete	cured

20	>16	>16	256	128	indifference	cured

21	>16	>16	128	256	complete	relapsed

**MIC50/90**			**256/512**	**256/256**		

**Table 4:** Association between meropenem actual MIC (obtained with macrobroth dilution) and clinical outcome after stratification according to meropenem MIC.

**Actual MIC meropenem (** ***μ*** **g/mL)**	**Subjects, n (%)**	**Outcome at 60-days, n (%)**
32	1 (5)	Cure: 1/1 (100)

128	6 (30)	Cure: 4/6 (66.7)
		Relapse: 2/6 (33.3)

256	8 (40)	Cure: 6/8 (75)
		Death: 2/8 (25)

512	5 (25)	Cure: 5/5 (100)

## Data Availability

The data used to support the findings of this study are available from the corresponding author upon request.

## Abbreviations

CSE: Carbapenem-susceptible* Enterobacteriaceae*
CP-CRE: Carbapenemase producing carbapenem-resistant* Enterobacteriaceae*
DC: Double-carbapenem
BAT: Best available treatment
MIC: Minimal inhibitory concentration
MEM: Meropenem
ETP: Ertapenem.
